# Quality of selected anti-retroviral medicines: Tanzania Mainland market as a case study

**DOI:** 10.1186/s40360-021-00514-w

**Published:** 2021-08-26

**Authors:** Sophia Mziray, Betty A. Maganda, Kissa Mwamwitwa, Adam M. Fimbo, Seth Kisenge, Gerald Sambu, Yonah H. Mwalwisi, Adonis Bitegeko, Emmanuel Alphonce, Akida Khea, Danstan H. Shewiyo, Eliangiringa Kaale

**Affiliations:** 1 Tanzania Medicines and Medical Devices Authority, P.O BOX 75150, Dar es salaam, Tanzania; 2 Department of Pharmaceutics and Pharmacy Practice, Muhimbili University of Health and Allied Sciences, P.O BOX 65013, Dar es salaam, Tanzania; 3 Pharm R&D Lab, School of Pharmacy, Muhimbili University of Health and Allied Sciences, P.O BOX 65013, Dar es salaam, Tanzania; 4 Department of Medicinal Chemistry, Muhimbili University of Health and Allied Sciences, P.O BOX 65013, Dar es salaam, Tanzania

**Keywords:** Medicines quality, Substandard, Falsified, Post market surveillance, Antiretroviral medicines, Patient information leaflet

## Abstract

**Background:**

Antiretroviral drugs (ARVs) have significantly reduced morbidity, mortality and improved the quality of life of people living with HIV infection. Poor quality ARVs may result in harmful consequences such as adverse drug reactions, treatment failure and development of drug resistant strains and sometimes death, which in turn may undermine the healthcare delivery system. To ensure optimal treatment outcomes, medicines quality control must be undertaken regularly. This study was aimed at evaluating the quality of ARVs circulating on the Tanzania Mainland market.

**Methods:**

This was a survey study. ARVs samples were collected in 20 regions of Tanzania Mainland, between 2012 and 2018. All sampled ARVs were subjected to screening testing using the Global Pharma Health Fund® Mini-Lab kits. Sampled ARV’s that failed screening test or yielded doubtful results and 10 % (10 %) of all that complied with the screening test requirements were selected for full quality control testing. Quality control testing was conducted at the Tanzania Medicines and Medical Devices Authority (TMDA) laboratory a World Health Organisation prequalified. Samples collected from the medicine distribution outlets were also, subjected to product information review.

**Results:**

A total of 2,630 samples were collected, of which 83.7 % (2200/2630) were from port of entry (POEs). All sampled ARVs were screened and conformed to the specifications, except of the fixed dose combination (FDC) lopinavir/ritonavir 0.27 % (7/2630) and lamivudine/zidovudine/nevirapine 0.27 % (7/2630) that failed the disintegration test. Out of the 100 samples selected for full quality control testing, 3 % of them failed to comply with the specifications, of which FDC stavudine/lamivudine/nevirapine failed disintegration and assay tests 2 % (2/100) and 1 % (1/100), respectively. Samples failing the assay test had low content of stavudine (86.6 %) versus specification limits (90 -110 %). Out of the 430 samples which were subjected to product information review, 25.6 % (110/430) failed to comply with the TMDA packaging and labelling requirements.

**Conclusions:**

The quality of majority of ARVs circulating on the Tanzania Mainland market was good, even so, significant deficiencies on labelling and packaging were observed. These results call for continuous monitoring of quality of medicines circulating on the Tanzania Mainland market.

## Background

Human immunodeficiency virus (HIV) infection and acquired immunodeficiency syndrome (AIDS) are still major global health problem particularly in sub-Saharan Africa [1, 2]. Globally, HIV/AIDS has remained one of the leading causes of morbidity in the last three decade [3–5]. The prevalence of HIV/AIDS amongst Tanzanian aged between 15 and 64 years is 5 % [1, 4]. According to UNAIDS and World Health Organization (WHO) reports, 21.7 million HIV-infected patients are currently, accessing antiretroviral therapy (ART) worldwide [1–3], with about 1.2 million of them residing in Tanzania [4]. The ART have significantly reduced morbidity and mortality, by preventing relapse and opportunistic infections and has lowered the risk of HIV transmission [6, 7]. Specifications to measure the quality of medicines are defined by manufacturers. Thus, medicines quality, are built into medicines during product development and production [8].

Even so, poor quality medicines are global problem, particularly in low and middle income countries. It is estimated that 10.5 to 50 % of the medicines circulating on markets of low and middle income countries are either falsified or substandard, of which ARVs are of no exception [8–23]. Existence of a significant number of porous borders in these countries may accounts for their vulnerability to the infiltration of falsified and substandard medicines.

In 2003 and 2007, falsified ARVs were found circulating in the informal market of Democratic Republic of Congo and Zimbabwe, respectively [14, 24]. A falsified triple antiretroviral combination product, Ginovir 3D was also reported in Côte d’Ivoire in 2003 [14]. Kenya and Zimbabwe, in 2011 and 2013, respectively reported availability of falsified ARVs in their informal market [25, 26]. Again, in 2012 about 12,000 bottles of falsified ARVs were recalled from Tanzania market [27]. All tested indinavir samples had high content (112.6 -118 %) of the claimed active pharmaceutical ingredient (API) compared to the official limit of 95–105 % in a study conducted in Tanzania [24]. Equally, in that study one sample of stavudine failed the dissolution test, by releasing only 56 % of API versus the specified 80 % [24]. In another study conducted by the WHO in seven African countries 1.8 % of the tested ARVs samples failed to meet compendia specifications [21]. Poor quality ART poses a major public health threat such as treatment failure, drug toxicity, poor diseases prognosis and emergence of resistant strains; increased co-morbidities and healthcare cost to government and individual and sometimes may cause death [8, 21, 22, 28]. Of which in return leads to loss of public confidence in the health care delivery system.

Recognizing public health consequences as a result of substandard and falsified medicines, national regulatory bodies should continued monitoring quality of medicines circulating in the legal market.

This study was aimed at evaluating the quality of selected ARVs circulating on the Tanzania Mainland market. The primary objective of this study was to determine the proportion of poor quality ART circulating on the market and secondary was to determine the quality of information on the primary and secondary packaging and availability of patient information leaflet.

## Methods

### Study design

This was a survey, cross sectional study, part of the continuous Post Market Surveillance (PMS) Country Program assessing the quality of antiretroviral, anti-tuberculosis and anti-malarial medicines in Tanzania.

### Study settings and duration

This study was conducted in 20 out of 26 regions of Tanzania mainland namely; Morogoro, Tanga, Dar es Salaam, Dodoma, Coast, Simiyu, Njombe, Arusha, Shinyanga, Kagera, Singida, Rukwa, Iringa, Geita, Kilimanjaro, Mara, Mbeya, Mtwara, Mwanza and Ruvuma. The regions were selected based on the pre-defined criteria including, highly populated regions, regions bordering other countries, regions with high prevalence of HIV infection and those reported to have medicine quality problems. Samples were collected from medicines distribution outlets between 2012 and 2015 and port of entries between 2012 and 2018.

### ARVs sampling site and sample collection

During the surveillance convenience sampling method was used for the collection of the selected ARVs. A normal inspection procedure without informing the outlet was used in sample collections process. The ARVs samples were collected from two distribution levels:-.

- Level I: The highest distribution of the distribution system which covered the port of entries (POEs) and Medical Stores Department (MSD).
- Level II: This covered public and private hospitals, dispensaries, health centres, retail and wholesale pharmacies and Accredited Drug Distribution Outlets (ADDO).

Collection sample sites were selected in such a way to cover urban and sub urban areas and all distribution levels. Systematic random sampling technique was used in the selection of outlets per region.

Samples were collected by trained drug inspectors according to the prepared sampling plan and TMDA standard operating procedures. At the collection site, 2 batches were sampled per product and one batch per brand of the targeted solid dosage form and strength. In this survey, not less than 100 tablets were collected per individual sample.

All samples were collected in their original containers. Each collected sample was coded for traceability. Sample code included brand name, region, sampling site and sampling date. The following information were recorded for each sample in the Sample Information Collection Form: Brand and generic name, dosage form, strength, batch or lot number, date of manufacturing and expiration, name and address of the manufacturer, country of manufacturer, TMDA registration number, packaging and pack size, availability of package information leaflet, language and storage instruction, physical appearance of the primary and secondary package, site and date of sample collection.

Coded samples with their respective Sample Information Collection Form were kept in the labelled sampling envelopes and sealed.

Storage and handling of samples during collection, transportation and before analysis complied with the manufacturer’s instructions.

All collected samples were subjected to screening test at TMDA zone laboratories and respective quality assurance (QA) centres. Those from medicine distribution outlets were further subjected to product information review (PIR).

Of the sampled ARVs, 100 % of those that did not comply with screening testing requirements or yielded doubtful results and 10 % of those that complied with screening test requirements were dispatched to TMDA-WHO prequalified laboratory for full quality control analysis.

### ARVs selection criteria

ARVs selected in this surveillance were those frequently prescribed for the management of HIV-infection with main focus on first line regimens as per National Guidelines for Management of HIV/AIDS [29] or those reported to have quality problem. These included the following monocomponent: efavirenz, nevirapine, lamivudine, zidovudine, abacavir sulphate, tenofovir disoproxil fumarate and fixed dose combination (FDC) containing tenofovir disoproxil fumarate /emtricitabine, lamivudine/zidovudine/nevirapine, lamivudine/stavudine/nevirapine, tenofovir/lamivudine/efavirenz, tenofovir/ emtricitabine/efavirenz and lopinavir/ritonavir.

### Quality evaluation

#### Product information review

Prior to further laboratory analysis, samples collected from medicine distribution outlets were subjected to PIR. Each collected sample was checked for information on the primary and secondary packaging, availability and information on package information leaflets (PIL) against TMDA labelling requirements and approved product information. Parameters checked during PIR included but not limited to product’s brand and generic name, dosage form and strength, name and address of the manufacturer, batch or lot number, date of manufacturing and expiration, TMDA registration number, packaging and pack size, indication, warnings, and precautions, language and storage instructions. The information was recorded on a standardized form.

#### Tier I laboratory screening testing

All samples were subjected to screening test using Global Pharma Health Fund^®^ (GPHF) Mini-Lab kits manual [30]. The performed tests were simple visual inspection, disintegration and identification test using Thin Layer Chromatography (TLC) method.

##### Visual inspection

All sampled solid dosage forms were checked for shape uniformity, physical damage (broken, erosion, crack, capping and chipping), altered surface (coating swelling), odor, discoloration and dirty marks.

##### Simple disintegration test

A simple disintegration test was performed to assess the possibility of instant-release of oral solid dosage forms as described in the GPHF Mini-Lab kits manual [30]. The sample was considered failed if did not disintegrate within 30 min in three (3) consecutive independent tests.

##### Product identification by thin layer chromatograph

This method was used for product identification and qualitative determination of active ingredients, related substances and impurities present in the dosage forms. This method employed the principle of comparing spots test sample and reference solutions according to the GPHF Mini-Lab kits manual. The principal spot obtained with the test sample solution was required to correspond with the chromatographic runs of the standard solution in terms of colour, shape, size, intensity and retardation factor (R_f_) value. The test sample was considered failed if the R_f_ value of the test sample was different by more than 10 % from that of the standard sample and if the intensity of the spot was less than that of a reference containing 80 % of the stated amount of the API. This had to be observed in three independent experiments.

#### Tier II laboratory confirmatory testing

All samples collected from medicines distribution outlets that had failed screening test, 10 % of those which had passed screening test, and those with doubtful results were subjected to confirmatory testing. Confirmatory testing was carried out at the TMDA-WHO prequalified laboratory as per United State Pharmacopoeia (USP) monograph requirements [31] or manufacturer’s methods and/or in house specifications where no official pharmacopoeia monograph existed.

Typical parameters tested were physical appearance, identification, disintegration, dissolution, assay, related substances and weight uniformity.

#### Data management and statistical analysis

The collected data were checked for any inconsistencies. The data was double-entered into a Microsoft Access database, verified and exported to SPSS (version 20) software for analysis. The SPSS was used only in the analysis of the dataset of PIR for the sampled ARVs. Descriptive statistics was used wherever appropriate and results are presented as frequencies and percentage.

## Results

### Antiretroviral samples collected

**Fig. 1:**
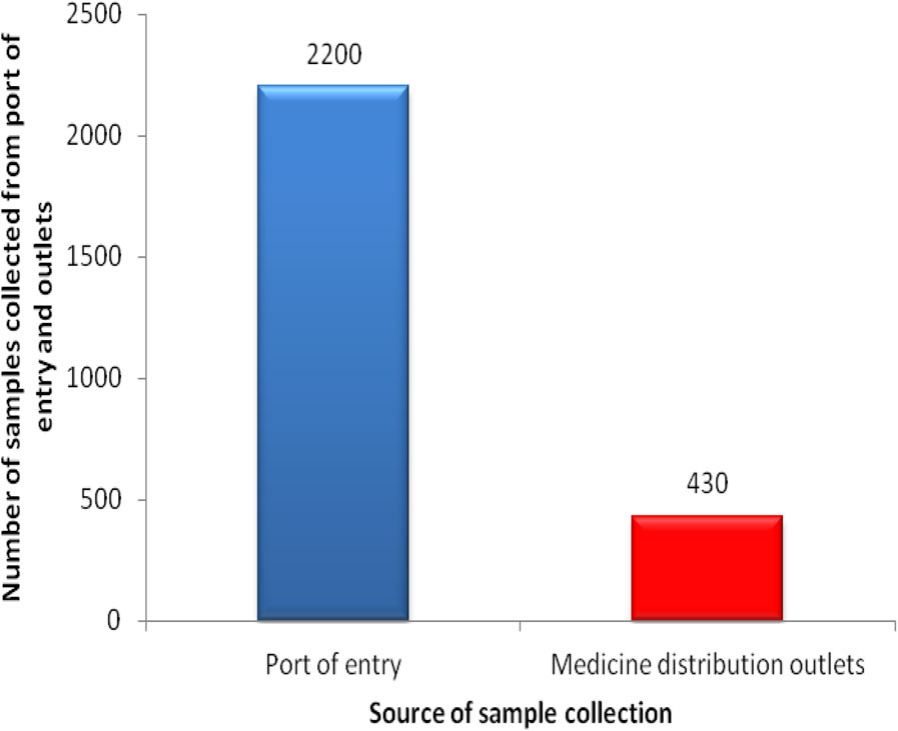
Proportion of antiretroviral drug samples collected from port of entry and medicine distribution outlets

A total of 2,630 ARVs samples were collected between 2012 and 2018 from POEs and medicine distribution outlets in Tanzania Mainland. Large quantity of samples 83.7 % (2,200/2,630) were collected from POEs as depicted in Fig. 1.

**Fig. 2:**
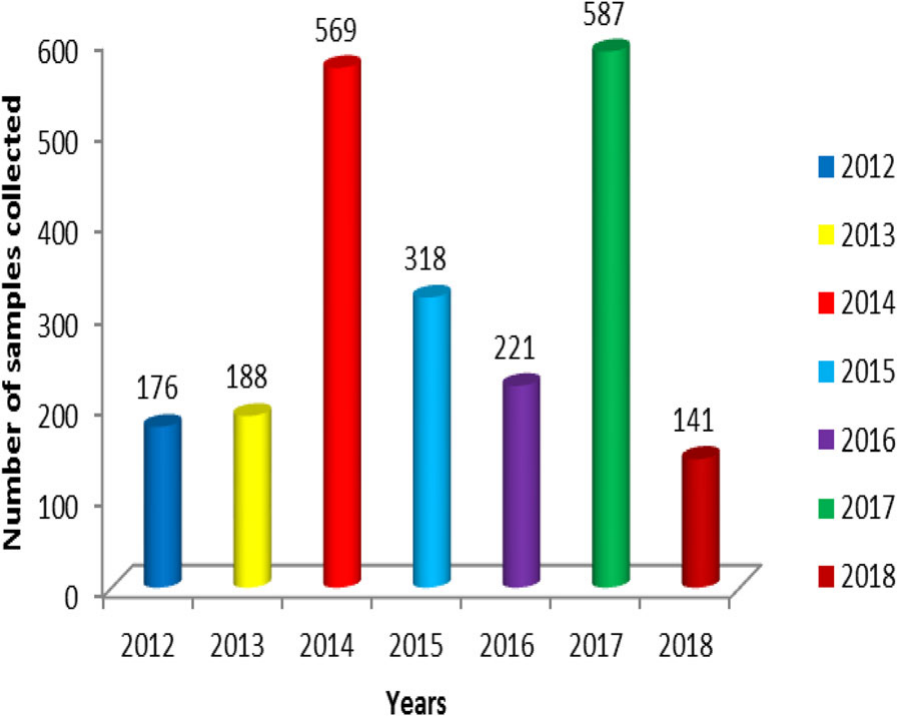
Number of antiretroviral drug samples collected at the port of entry annually (2012–2018)

ARVs samples from POEs were collected in large quantity in the years 2014, 2015, 2016 and 2017, 77 % (1,695/2,200) compared to other years, as shown in Fig. 2.

**Fig. 3:**
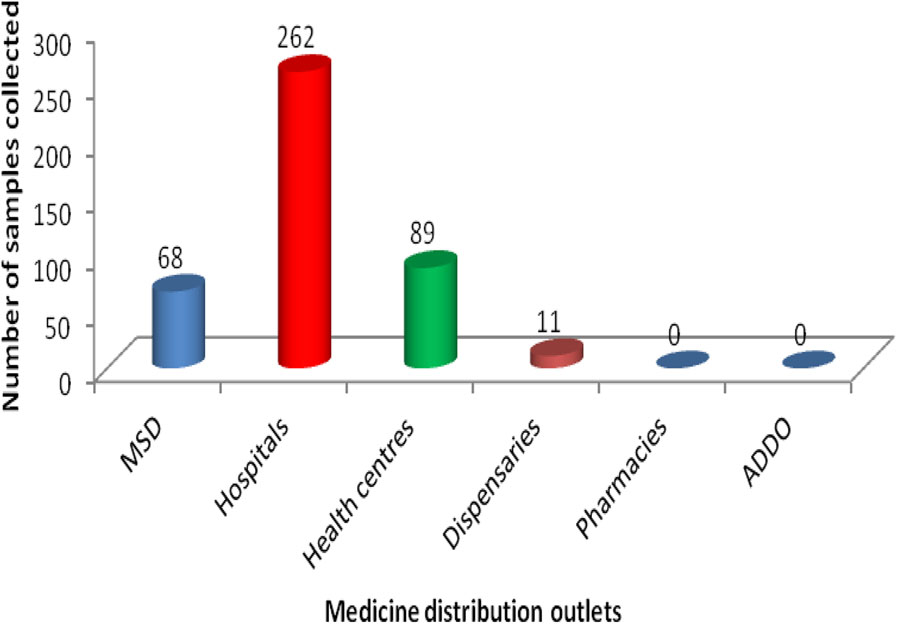
Samples of antiretroviral drugs collected from different medicine distribution outlets

Regarding, the number of samples from different medicine distribution outlets, hospitals ranked highest amounting to 61 % (262/430) as portrayed in Fig. 3. Regional wise, 30 % (129/430) were sampled from Dar es Salaam, Mwanza and Iringa and least quantity 1.4 % (6/430) was collected from Kagera.

Thirteen generic brands (6 API and 7 fixed dose combinations) contributed to the 430 samples collected from the medicine distribution outlets. The FDC of lamivudine/zidovune/nevirapine tablets was collected in large quantity 17 % (73/430) followed by lamivudine/zidovudine tablets combination 16.3 % (70/430) as compared to other ARVs.

### Quality evaluation

#### Product information review

**Fig. 4:**
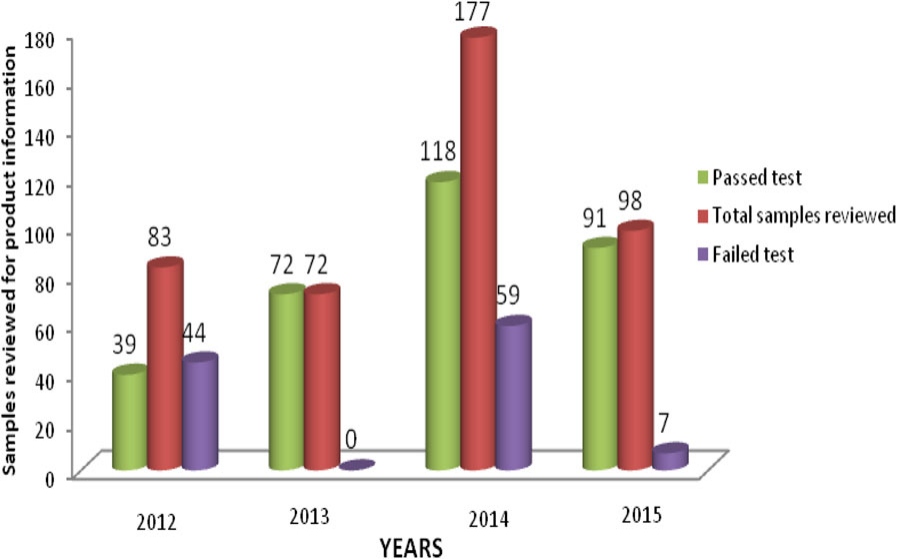
Results of product information review per year

In this study only samples collected from medicine distribution outlets were subjected to PIR and the results are summarized in Fig. 4. Of the examined samples, 25.6 % (110/430) did not comply with the packaging information requirements. The highest failure rate was found in the samples collected in the year 2012, 53 % (44/83). Interesting observation was found in the year 2013, whereas, all samples passed PIR assessment. Inappropriate or lack of storage condition, lack of the name and address of the manufacturer, discrepancy of address of the manufacturer on the primary and secondary packaging and lack of package insert were the common deficiencies. On the other hand, all sampled medicines were found to be registered in Tanzania, but some of labels did not indicate the Tanzania registration numbers. The trend is comparable to recent studies conducted on antimalarial and antihypertensive medicines [9, 10].

#### Laboratory tier I screening testing

All ARVs, sampled from POEs conformed to tier I screening test requirements. All sampled ARVs from medicine distribution outlets conformed to visual appearance and identification test requirements. However, 3.3 % (14/430) of the samples from the medicines distribution outlets, failed disintegration test; these were FDC of lopinavir/ritonavir tablets (7/430) and lamivudine/zidovudine/nevirapine tablets (7/430).

#### Laboratory tier II confirmatory testing

Of the 430 ARVs samples collected from medicine distribution outlets and subjected to tier I screening testing, 100 of them were subjected to tier II confirmatory test. These samples included all those failed screening tests and with doubtful results and10 % of those which passed screening test as depicted in Table 1.

**Table 1:** Sampled antiretroviral medicines from medicine distribution outlets and number of samples selected and tested for confirmatory testing

Product name	Samples collected and screened			Confirmatory test	
	**Samples collected**	**Samples screened and passed**	**Samples Failed**	**Samples eligible for confirmatory**	**Samples selected and tested**
Efavirenz	66	66	0	7	14
Nevirapine tablets	34	34	0	4	7
Lamivudine tablets	10	10	0	1	10
Zidovudine tablets	2	2	0	1	2
Tenofovir Disoproxil Fumarate tablets	5	5	0	1	5
Lamivudine/Zidovudine tablets	70	70	0	7	7
Abacavir Sulphate tablets	16	16	0	2	4
Lopinavir/Ritonavir tablets	18	12	7	9	9
Tenofovir Disoproxil Fumarate /Emtricitabine tablets	14	14	0	2	4
Tenofovir Disoproxil Fumarate /Lamivudine/Efavirenz tablets	29	29	0	3	6
Lamivudine/Zidovune/Nevirapine tablets	73	73	**7**	14	14
Lamivudine/Stavudine/Nevirapine tablets	66	59	0	10	10
Tenofovir Disoproxil Fumarate /Efavirenz/Emtricitabine tablets	27	27	0	3	8
**Total**	**430**	**416**	**14**	**61**	**100**

**Fig. 5:**
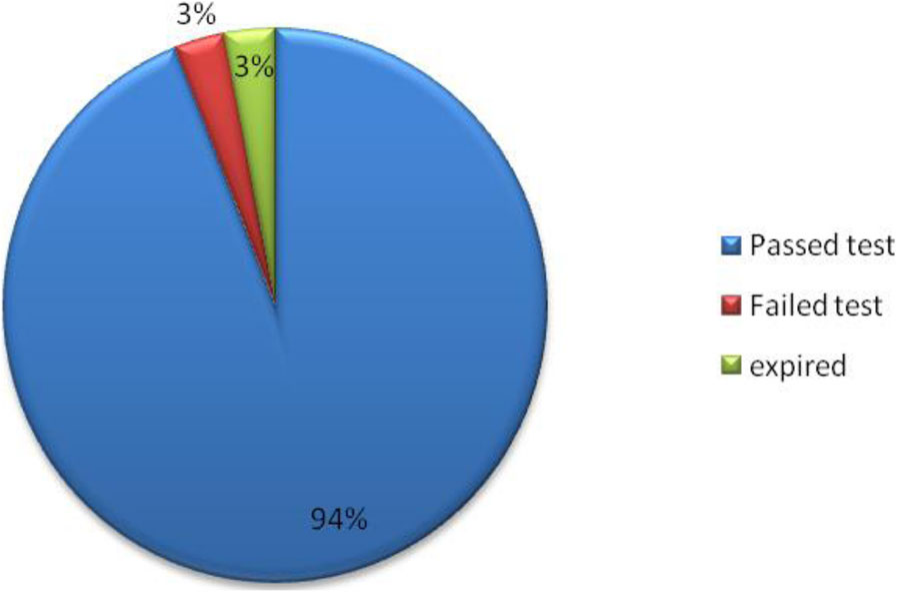
Proportion of samples passed laboratory tier II confirmatory testing

Majority of samples 94 % (94/100) complied with confirmatory test requirements. However, 3 % (3/100) failed confirmatory test and 3 % (3/100) samples were beyond the expiration dates at the time of analysis, hence, were not analyzed. The failed samples were of FDC of stavudine/lamivudine/nevirapine 3 % (3/100) which failed disintegration test 2 % (2/100) and assay test 1 % (1/100), having low content of stavudine (86.6 %) of the specified amount (limit 90 -110 %). This necessitated more samples from the same manufacturer to be subjected to confirmatory test as described in the USP. The additional samples also failed assay test with results ranging between 86.4 and 87.1 %. This confirmed the non conformity of stavudine against the specified requirements. These results are summarized in Fig. 5.

## Discussion

Quality medicines are essential to promote public health and disease management. Still, various studies conducted worldwide, have portrayed availability of poor quality medicines in the market including ARVs, mostly in developing countries such as Tanzania [8–23].

This survey assessed the quality of 2,630 ARVs samples, collected from medicine distribution outlets and POEs. A total of twenty (20) regions representing more than 76 % of the regions of Tanzania Mainland were selected for the purpose. The selection of the regions to be surveyed included some regions bordering other countries; frequency of inspection by the regulatory bodies; areas previously reported to have medicines quality problems and HIV prevalence patterns as indicated in HIV/AIDS and malaria indicator survey of 2011/12 and HIV impact survey of 2016/17 [28, 29]. In this survey, 30 % (129/430) of samples from medicine distribution outlets were collected in three regions namely Mwanza, Dar es Salaam and Iringa matching with high prevalence of HIV infection [4, 29].

It is clear from these results that, majority 83.7 % (2200/2630) of the samples were collected from POEs showing the dependence of the country on imported medicines. This observation is in agreement with TMDA data of 2017 [32] and well supported with results from this study that among of the collected samples none were locally produced.

A significant increase of ARVs sampled from the POEs in the year 2014, 2015, 2016 and 2017 compared to 2012/13 can be associated with the scaling up of ARVs access in low and middle income countries through the WHO and Global Fund initiatives [33]. Improved access to ARVs has accounted to the levelling off of new HIV infections cases, deaths and life longevity amongst HIV-infected patients [1–3].

Despite the inclusion of ADDO and pharmacies among the medicine distribution outlets, no sample was obtained from these sources. This is an interesting finding on compliance to national and international requirements on availability of ARVs in specialized HIV-clinics, hospitals, health centres and dispensaries [33].

Among the collected FDC samples, oral solid dosage forms were the majority (99.8 %). FDCs are desirable and recommended by the WHO, as it simplify treatment, ideally resulting in improved medication concordance, clinical outcomes and quality of life of patients [7]. The FDC of lamivudine/zidovudine/nevirapine ranked highest (17 %) amongst other ARVs. This was a default regimen during the survey period which is reflected by its wide collection [29].

Samples collected from the POEs were not subjected to PIR. This is because, they were being collected on daily basis within the framework of medicines quality assurance system in which the first step is laboratory screening.

On the other hand, 25.6 % (110/430) of the sampled ARVs from medicines distribution outlets subjected to PIR failed to comply with requirements. The failure rate was highest in 2012, but decreased significantly in subsequent years, as shown in Fig. 3. Adherence to regulatory requirements of individual countries as well as harmonization efforts on technical requirements across the regions could explain the continuing improvement. Compared with PIR results for other medicine categories in the implementation of PMS programs, these results are far much better [9, 10, 34]. Even so, results obtained from a study conducted in Cameroon are much far better compared to PIR results of this survey [35].

All pharmaceutical products must be stored under conditions provided by manufacturers to ensure that their potency and qualities are not compromised during the distribution process and storage. In this study it was observed that some of the manufacturers did not indicate proper storage conditions while some provided conditions that are not achievable in tropical set up, such as “store below 25^o^C”. In country like Tanzania which fall under ICH zone IVb climatic condition it is not easy to achieve/maintain the aforementioned storage condition [36]. Improper storage of pharmaceutical products could negatively impact on their potency, quality, efficacy, safety and subsequently compromising the quality of life of the end users [37, 38].

Information on package inserts is necessary since, it is associated with drug safety, adherence to treatment, rational drug use and reporting adverse drug reactions [39]. Our findings revealed absence of package inserts from majority of the collected samples.

All ARVs sampled in this study were registered in Tanzania but the registration number which is a prerequisite was not indicated on the labels to some packages. The Tanzania registration number enables easy identification of pharmaceutical products authorized by TMDA to be in the market and prevents availability of falsified products on the market.

All sampled ARVs, conformed to tier I screening test specifications except 0.53 % (14/ 2630 of FDC of lamivudine/zidovudine/nevirapine and lopinavir/ritonavir tablets which failed disintegration test. This finding is consistent with previous results from a survey study which was conducted in seven African countries to determine the quality of ARVs circulating on their market [20]. Passing Tier II confirmatory test for the aforementioned samples provided evidence that were of good quality. The situation which stresses on the need for confirmatory tests before conclusion can be made on quality of medicines.

In confirmatory testing by full monograph, all samples tested met the specifications except two (2) samples of FDC of stavudine/lamivudine/nevirapine which failed disintegration test and one (1) sample of the same which failed assay test. A sample which failed the assay specifications had stavudine content below the acceptance criteria. This was confirmed by the consistency of results obtained from repeated analysis done in triplicate. The cause of failure in the screening test for only products sampled from medicine distribution outlets may be due to poor storage conditions. Improper storage of pharmaceutical products is known to decrease their potency and quality [37, 38, 40].

As a point of information, the stavudine concentration is no longer an issue of discussion since ARVs regimen containing stavudine has been phased out in the country due to its severe toxicity [41–43].

Disintegration of tablets within 30 min predicts dissolution within the required time *in vivo* [44], and is a precondition to drug absorption process. Poor absorption of the medicine is associated with poor therapeutic outcomes and development of resistant strains. Disintegration failure rate in this survey was only 2 %. This finding is comparable to previous finding reported by the WHO from a study conducted in seven African countries, of the tested samples only 0.6 % failed disintegration test [20]. Still, this cannot be ignored because presence of substandard drugs in the market at any percentage poses threats to the public. These threats include but not limited to poor treatment outcomes and disease prognosis, drug resistance and death but as well in terms of economic implications and effects on global pandemics [37, 38].

Results for assay test in this study are consistent with reports from various studies worldwide, which have shown that failure rates for ARVs, is very low [2, 20, 21, 35, 45]. Only 0.25 % of ARVs, sampled in seven African countries failed the assay test and none of them failed in the study conducted in Cameroon [20, 35]. This can be explained by the fact that most of ARVs in the market are procured by government agencies with donor funding. The funding agencies impose stringent procurement condition such as requirements for the WHO pre-qualification for all suppliers and so, low failure rates [20, 46]. Low failure rates too have been observed in countries such as Tanzania using WHO-prequalified products as reported in a previous study [20].

Of importance is that for all sampled medicines that were deemed to have deficiencies during PIR, the TMDA directed manufacturers to bring request for amendments for all deficiencies observed. Also, the manufacturers and local agents were informed to recall all batches that failed the confirmatory test from the market. The market authorization holders were further directed to halt importation of such products and to conduct thorough investigations to identify possible causes of the failure.

## Conclusions

The quality of the majority of ARVs surveyed was good, however, significant deficiencies on labelling and packaging were observed. These results indicates that the efforts made by the WHO and other organizations on prequalification and purchase policies on ARVs have a positive effect. All the same, call for continuous monitoring of quality of medicines circulating on the Tanzanian market is emphasized.

## Data Availability

All relevant data generated and analyzed during this study are available from the Director General of TMDA; email: adam.fimbo@tmda,go.tz on reasonable request.

## Abbreviations

ADDO: Accredited Drug Dispensing Outlet
MSD: Medical Stores Department
TMDA: Tanzania Medicines and Medical Devices Authority
TLC: Thin Layer Chromatography
ARVs: Antiretroviral drugs
WHO: World Health Organization
POEs: Port of entries
